# Effects of the potential lithium-mimetic, ebselen, on brain neurochemistry: a magnetic resonance spectroscopy study at 7 tesla

**DOI:** 10.1007/s00213-015-4189-2

**Published:** 2016-01-12

**Authors:** Charles Masaki, Ann L. Sharpley, Beata R. Godlewska, Adam Berrington, Tasuku Hashimoto, Nisha Singh, Sridhar R. Vasudevan, Uzay E. Emir, Grant C. Churchill, Philip J. Cowen

**Affiliations:** Department of Psychiatry, Warneford Hospital, University of Oxford, Oxford, OX3 7JX UK; Department of Pharmacology, University of Oxford, Mansfield Road, Oxford, OX1 3QT UK; The Oxford Centre for Functional MRI of the Brain, Nuffield Department of Clinical Neurosciences, John Radcliffe Hospital, University of Oxford, Oxford, OX3 9DU UK; Current Address: Centre for Neuroimaging Studies, PO 089, De Crespigny Park, London, SE5 8AF UK

**Keywords:** Ebselen, Bipolar disorder, Inositol, Glutamate, Magnetic resonance spectroscopy

## Abstract

**Rationale:**

Lithium is an effective treatment for bipolar disorder, but safety issues complicate its clinical use. The antioxidant drug, ebselen, may be a possible lithium-mimetic based on its ability to inhibit inositol monophosphatase (IMPase), an action which it shares with lithium.

**Objectives:**

Our primary aim was to determine whether ebselen lowered levels of inositol in the human brain. We also assessed the effect of ebselen on other brain neurometabolites, including glutathione, glutamate, glutamine, and glutamate + glutamine (Glx)

**Methods:**

Twenty healthy volunteers were tested on two occasions receiving either ebselen (3600 mg over 24 h) or identical placebo in a double-blind, random-order, crossover design. Two hours after the final dose of ebselen/placebo, participants underwent proton magnetic resonance spectroscopy (^1^H MRS) at 7 tesla (T) with voxels placed in the anterior cingulate and occipital cortex. Neurometabolite levels were calculated using an unsuppressed water signal as a reference and corrected for individual cerebrospinal fluid content in the voxel.

**Results:**

Ebselen produced no effect on neurometabolite levels in the occipital cortex. In the anterior cingulate cortex, ebselen lowered concentrations of inositol (*p* = 0.028, Cohen’s *d* = 0.60) as well as those of glutathione (*p* = 0.033, *d* = 0.58), glutamine (*p* = 0.024, *d* = 0.62), glutamate (*p* = 0.01, *d* = 0.73), and Glx (*p* = 0.001, *d* = 1.0).

**Conclusions:**

The study suggests that ebselen produces a functional inhibition of IMPase in the human brain. The effect of ebselen to lower glutamate is consistent with its reported ability to inhibit the enzyme, glutaminase. Ebselen may have potential as a repurposed treatment for bipolar disorder.

## Introduction

Six decades after its introduction as a treatment for acute mania, lithium remains the most efficacious treatment for bipolar disorder. As a prophylactic agent, lithium prevents both mania and depression and is the only psychotropic drug shown reliably to decrease suicidal behavior (Miura et al. 2014; Cipriani et al. 2013; Geddes et al. 2010). However, lithium treatment has several drawbacks including poor tolerance, a narrow therapeutic window, longer-term toxicity, particularly for the kidney, and the risk of teratogenicity (McKnight et al. 2012; Shine et al. 2015). Therefore, a form of drug treatment which has the efficacy of lithium without its toxicity would be a worthwhile development.

Rational design of a lithium-like mood stabilizer could be pursued based on its mechanism of action, but lithium’s therapeutic target remains unclear. Based on clinically relevant lithium concentrations (0.6–1.2 mM), the two most likely targets are glycogen synthase kinase 3 and inositol monophosphatase (IMPase) (Berridge et al. 1989; Belmaker et al. 1996; Agam et al. 2009). Recently, we reported inhibition of IMPase by ebselen (IC50 1.5 μM), a bioavailable antioxidant drug that has been tested in humans for other diseases including post-stroke neuroprotection and noise-induced hearing loss (Singh et al. 2013; Lynch and Kil 2009; Azad and Tomar 2014).

We found in animals that ebselen administration lowered brain myo-inositol levels, consistent with functional inhibition of IMPase (Singh et al. 2013), and subsequently in a healthy volunteer study, showed that three 600-mg doses of ebselen over 24 h lowered levels of myo-inositol in the anterior cingulate cortex but not in the occipital cortex as measured by magnetic resonance spectroscopy (MRS) at 3 T (Singh et al. 2015). The aim of the present study was to replicate this finding using a higher dose of ebselen and at a higher field strength (7 T). MRS at 7 T was chosen because the increase in signal to noise ratio (SNR) and spectral resolution allow for more precise metabolite quantification as well as the clear identification of separate glutamate and glutamine resonances as compared to 3 T (Tkáč et al. 2009). Assessment of the effects of ebselen on brain glutamate concentration is of interest because ebselen is reported to inhibit the glutamate-synthesizing enzyme, glutaminase, in vitro (Thomas et al. 2013).

## Methods

### Participants and study design

Ethical approval for the study was obtained from the National Research Ethics Service Committee (NRES), South-Central Oxford B. Twenty healthy volunteers (7 females, 13 males, mean age 25.1 years, range 20–38 years; mean BMI 22.7 kg/m^2^, range 18.7–30.0 kg/m^2^) were included in the study after giving full informed written consent. Exclusion criteria included a history of any DSM-IV Axis I psychiatric disorder (determined using the Standard Clinical Interview for Diagnostic and Statistical Manual for Mental Health Disorders—Fourth Edition), significant current medical condition, current regular medication (apart from the contraceptive pill), pregnancy or lactation, heavy smoking (defined as more than five cigarettes per day), having taken part in another study involving an investigational drug within the last 3 months, and contraindications to MRI scanning. Participants were asked to maintain stable exercise and diet as well as refrain from alcohol during study participation.

Ebselen capsules and identical matching placebo (containing microcrystalline cellulose) were purchased from Shasun pharmaceuticals Ltd. Participants were tested twice (7 days apart) receiving on one occasion ebselen and on the other, placebo in a random-order, double-blind, crossover design. Ebselen was administered in 6 × 200 mg capsules in three doses given over 2 days. On the day before the scan visit, participants were asked to take the first dose at 1 pm and the second dose at 10 pm. The final dose was taken 2 h prior to the MRI scan session. Participants were sent text message reminders a few minutes before they were due to take medication and were asked to confirm receiving the messages.

### Proton magnetic resonance spectroscopy

**Fig. 1:**
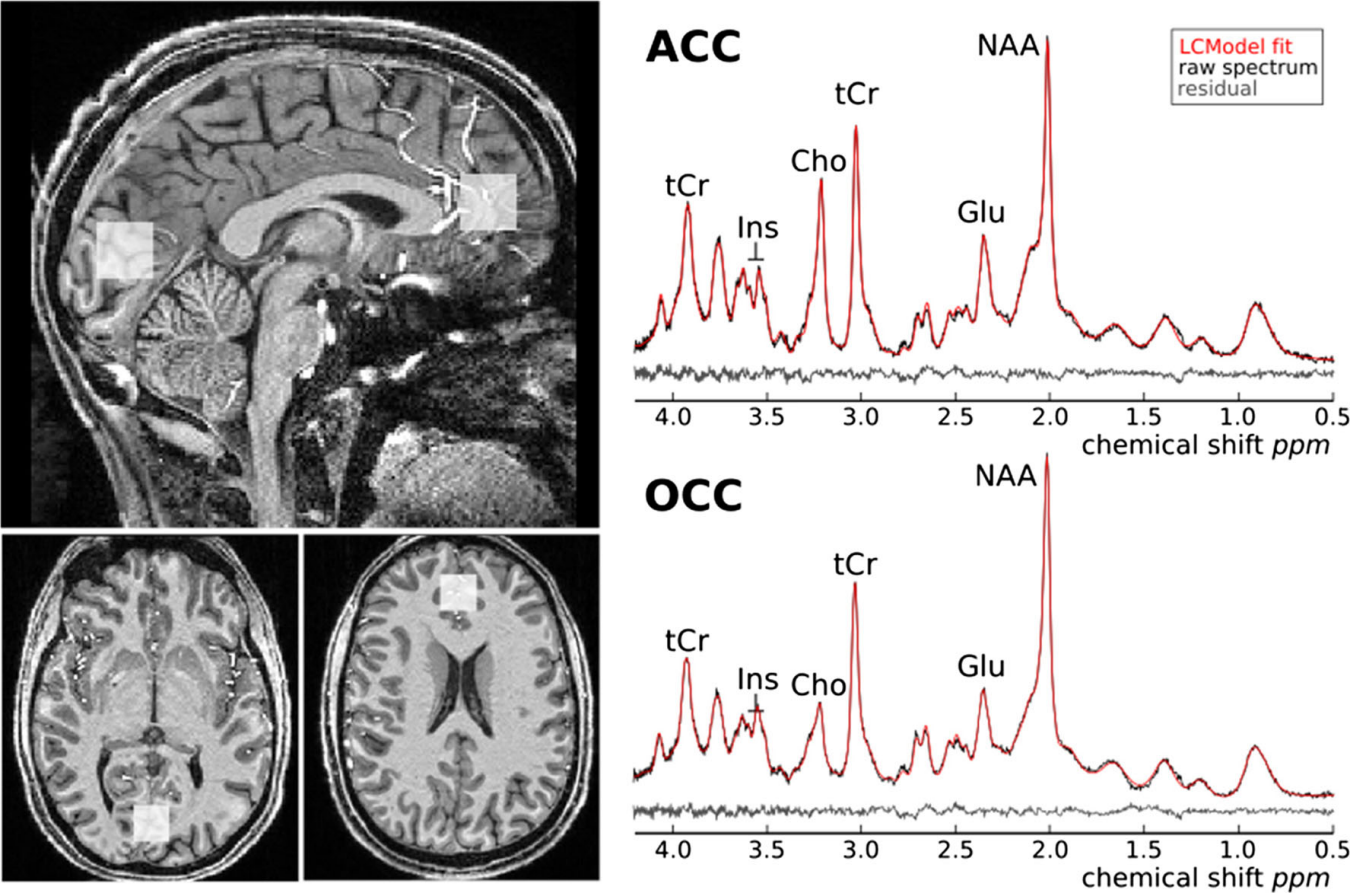
Voxel placement and representative spectra from the anterior cingulate cortex (ACC) and occipital cortex (OCC). Each acquired spectrum (64 averages) is overlaid with the metabolite fit from LCModel (*red line*) with major peaks labeled. The difference between the metabolite fit and underlying spectrum is shown below as a residual, which remains small and uniform indicating a high quality spectral fit. *tCR* total creatine, *Ins* myo-inositol, *Cho* choline, *Glu* glutamate, *NAA N*-acetylaspartate

Proton magnetic resonance spectroscopy (^1^H MRS) scanning took place at the Functional Magnetic Resonance Imaging of the Brain (FMRIB) Centre. Scanning was performed on a 7 T Siemens MAGNETOM scanner (Siemens, Erlangen, Germany) equipped with a Nova Medical 32 channel receive array head coil. Spectra were measured from two 8-ml voxels, one in the anterior cingulate cortex and the other in the occipital cortex (Fig. 1). Voxels were positioned manually by reference to 1-mm isotropic T1-MPRAGE image. To ensure reproducibility of voxel placement during both ^1^H MRS scan visits, screenshots of each anatomical region showing voxel placement in three planes were taken from each subject during the first visit. These were used to guide voxel placement during the second visit.

First- and second-order shims were first adjusted by gradient-echo shimming (Shah et al. 2009). The second step involved only fine adjustment of first order shims using FASTMAP (Gruetter and Tkáč 2000). Spectra were acquired using a Stimulated Echo Acquisition Mode (STEAM) pulse sequence (TE = 11 ms, TR = 5 s, number of transients = 64) with variable power radiofrequency pulses with optimized relaxation delay (VAPOR) water suppression and outer volume saturation (Emir et al. 2012). Unsuppressed water spectra acquired from the same voxel were used to remove residual eddy current effects and to reconstruct the phased array spectra.

Metabolites were quantified using LCModel (Provencher, 2001). The model spectra of aspartate (Asp), ascorbate/vitamin C (Asc), glycerophosphocholine (GPC), phosphocholine (PC), creatine (Cr), phosphocreatine (PCr), γ-aminobutyric acid (GABA), glucose (Glc), glutamine (Gln), glutamate (Glu), glutathione (GSH), myo-inositol (myo-Ins), *N*-acetylaspartate (NAA), *N*-acetylaspartylglutamate (NAAG), phosphoethanolamine (PE), scyllo-inositol (scyllo-Ins), and taurine (Tau) were generated based on previously reported chemical shifts and coupling constants (Govindaraju et al. 2000; Tkáč 2008) by using GAMMA/PyGAMMA simulation library of VeSPA for carrying out the density matrix formalism (VErsatile Simulation, Pulses and Analysis) (Soher et al. 2011). Simulations were performed with the same RF pulses and sequence timings as that on the 7 T system. A macromolecule spectrum acquired from the occipital cortex, using an inversion recovery sequence (TR = 3 s, TE = 11 ms, inversion time TI = 0.685 s), was included in the model spectra. Metabolite concentrations were obtained relative to an unsuppressed water spectrum acquired from the same VOI assuming a water content of 82 % for the occipital cortex and anterior cingulate, which primarily contain gray matter.

The MPRAGE images were segmented using FAST (FMRIB’s Automated Segmentation Tool, part of the FSL toolbox) to determine CSF fraction (fCSF) in the voxels (Zhang et al. 2001). Concentrations were then corrected for CSF fraction with the following formula: [Mcorr] = [M] · (1/[1 − fCSF]), where [Mcorr] = corrected concentration and [M] = metabolite concentration from LCModel output. Pairs of MRS spectra with a difference in full width at half-maximum (FWHM) difference of >0.01 ppm were excluded (three for the anterior cingulate cortex, none for the occipital cortex).

Metabolites quantified with Cramér-Rao lower bounds (CRLB, estimated error of the metabolite quantification) >50 % were classified as not detected. As a secondary filter to select reliable metabolite concentrations, only metabolites quantified with CRLB ≤50 % in at least half of the spectra from a brain region were reported. If the correlation between two metabolites was consistently high (correlation coefficient <−0.5) in a given region, their sum was reported, such as Glc + Tau, NAA + NAAG (tNAA, total NAA), Cr + PCr (tCr, total creatine), and GPC + PC (tCho, total choline).

### Statistics

Statistical analyses were performed in SPSS version 22. Differences in metabolite concentrations between placebo and ebselen administration were determined using separate multivariate analysis of variance (MANOVA) for the anterior cingulate cortex and occipital cortex. Significant effects on the MANOVA were followed up with post hoc paired sample *t* test. The change in myo-inositol concentration was taken as the primary end point.

## Results

The ebselen treatment was well tolerated and no participant dropped out of the study (the neuropsychological effects of ebselen treatment will be described in a separate report). MRS voxel placement and representative spectra from the anterior cingulate cortex (ACC) and occipital cortex (OCC) are shown in Fig. 1. For the ACC, we obtained 19 pairs of measurements (no measurements were obtained from one subject due to technical difficulty). Three pairs of spectra with an FWHM difference >0.01 ppm were excluded, resulting in 16 pairs being included in the analysis. For the OCC, all 20 pairs of measurements were included in the analysis. All the spectra were of high quality, with average signal to noise ratio (SNR) of 39.97 ± 1.04 (mean ± SEM), linewidth of 9.60 ± 0.34 Hz for the ACC and SNR of 45.65 ± 0.92, linewidth 9.62 ± 0.12 Hz for the OCC. All the metabolites of interest were quantified at an average CRLB of <15 %, consistent with high quality data at ultra high-field imaging.

**Fig. 2:**
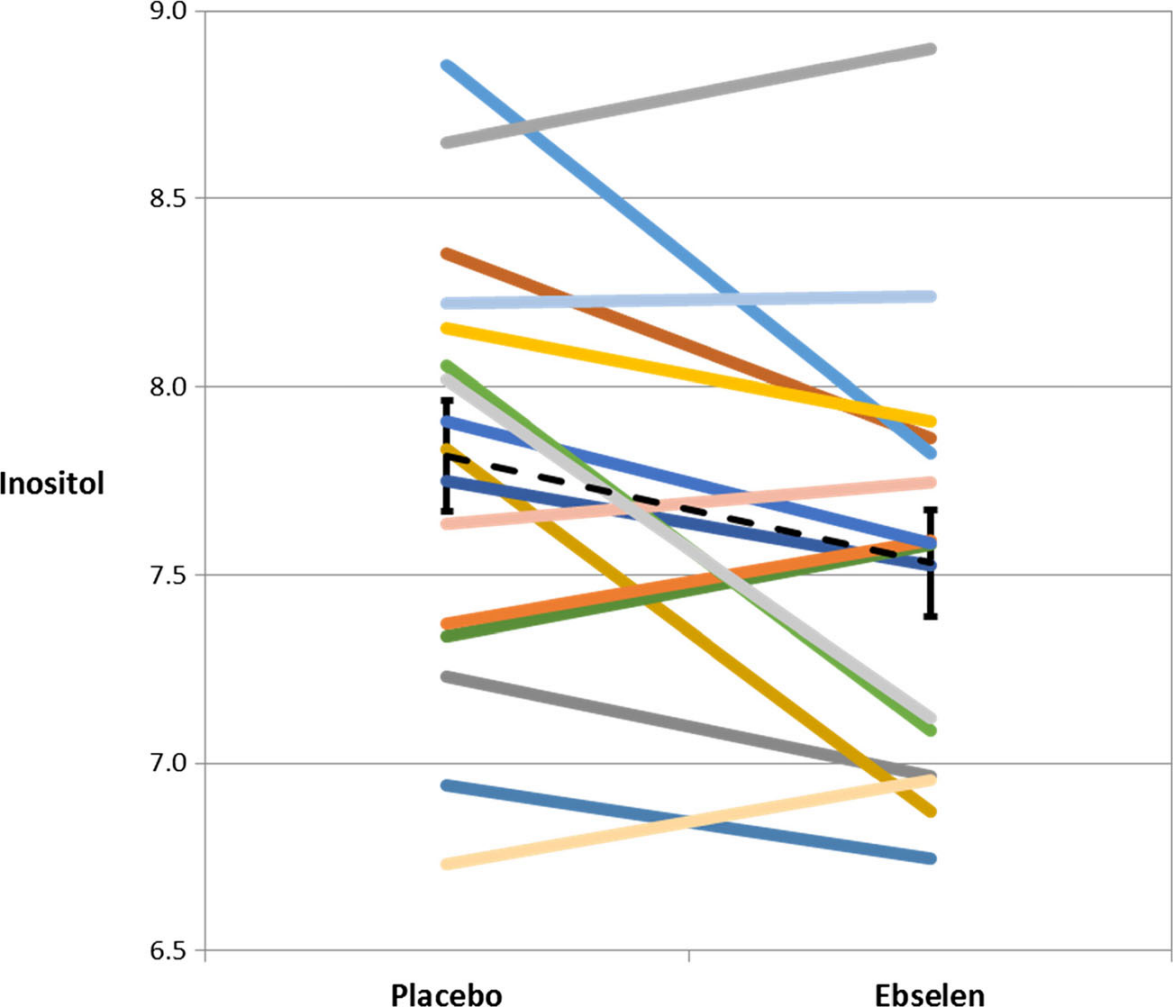
Anterior cingulate cortex concentrations of inositol (μmol/g) following treatment with ebselen (3600 mg over 24 h) or placebo in 16 individual subjects. Ebselen treatment resulted in a significant decrease in inositol (*p* = 0.028, paired *t* test). *Black dotted line* represents mean (and standard error) for group at each visit

**Fig. 3:**
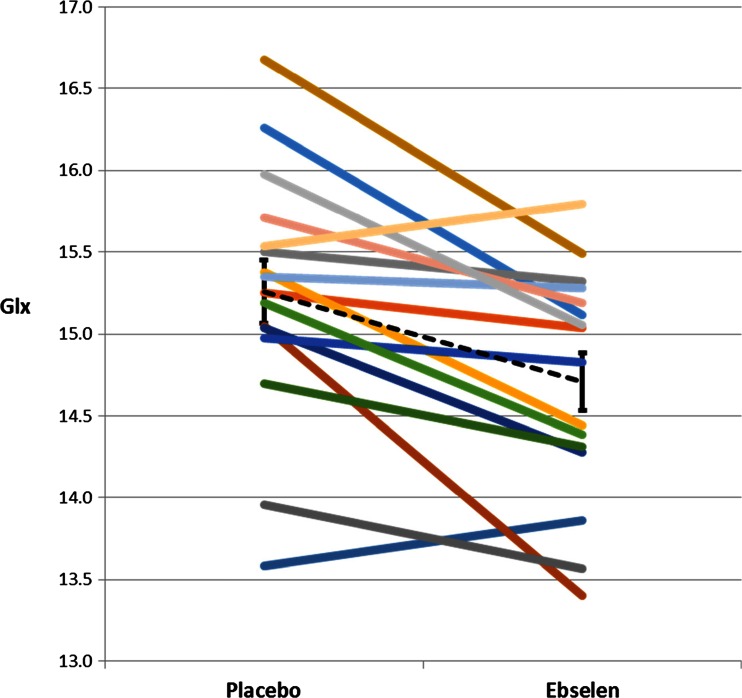
Anterior cingulate cortex concentrations of Glx (μmol/g) following treatment with ebselen (3600 mg over 24 h) or placebo in 16 individual subjects. Ebselen treatment resulted in a significant decrease in Glx (*p* = 0.001, paired *t* test). *Black dotted line* represents mean (and standard error) for group at each visit

The MANOVA for the anterior cingulate cortex (Wilks Lamda) showed a main effect of ebselen treatment (*F* = 12.48; *p* = 0.003) and a significant interaction between treatment and neurometabolite (*F* = 3.38; *p* = 0.044). Follow-up pairwise comparisons revealed that ebselen decreased inositol concentrations within this region (Table 1, Fig. 2). There were also significant reductions in glutathione, glutamine, glutamate, and Glx (Fig. 3), the latter being a composite of glutamate and glutamine. There was no change in concentrations of γ-aminobutyric acid (GABA) or total *N*-acetylaspartate (NAA) (Table 1).

**Table 1:** Absolute metabolite concentrations (μmol/g) given as mean ± SEM, in the anterior cingulate cortex following treatment with ebselen (3600 mg over 24 h) or placebo of inositol, *N*-acetylaspartate (NAA), glutathione (GSH), γ-aminobutyric acid (GABA), glutamate, glutamine, and Glx. The averages of the linewidth (Hz) and signal to noise ratio (SNR) have also been reported

	Placebo	Ebselen	Significance—paired *t* test
Inositol	7.82 ± 0.15	7.53 ± 0.14	0.028
NAA	10.53 ± 0.23	10.49 ± 0.26	0.789
GSH	1.31 ± 0.043	1.17 ± 0.07	0.033
GABA	2.04 ± 0.08	2.04 ± 0.07	0.984
Glutamate	11.66 ± 0.17	11.34 ± 0.15	0.010
Glutamine	3.60 ± 0.10	3.37 ± 0.10	0.024
Glx	15.26 ± 0.19	14.71 ± 0.18	0.001
Linewidth	9.66 ± 0.53	9.55 ± 0.43	0.743
SNR	39.5 ± 1.4	40.4 ± 1.6	0.264

The MANOVA for the occipital cortex (Wilks’ Lamda) showed neither a main effect of ebselen treatment (*F* = 0.01; *p* = 0.93) nor a significant interaction between treatment and neurometabolite (*F* = 0.99; *p* = 0.47) (Table 2).

**Table 2:** Absolute metabolite concentrations (μmol/g) given as mean ± SEM, in the occipital cortex following treatment with ebselen (3600 mg over 24 h) or placebo of inositol, *N*-acetyl-aspartate (NAA), glutathione (GSH), γ-aminobutyric acid (GABA), glutamate, glutamine, and Glx. The averages of the linewidth (in Hz) and signal to noise ratio (SNR) have also been reported

	Placebo	Ebselen	Significance—paired *t* test
Inositol	6.66 ± 0.14	6.69 ± 0.15	0.651
NAA	11.95 ± 0.17	11.88 ± 0.15	0.567
GSH	0.95 ± 0.03	0.93 ± 0.03	0.570
GABA	1.79 ± 0.07	1.85 ± 0.06	0.314
Glutamate	9.32 ± 0.14	9.27 ± 0.15	0.610
Glutamine	2.80 ± 0.08	2.83 ± 0.08	0.770
Glx	12.13 ± 0.17	12.10 ± 0.15	0.843
Linewidth	9.66 ± 0.17	9.58 ± 0.17	0.748
SNR	46.1 ± 1.3	45.3 ± 1.4	0.484

## Discussion

As in our previous MRS study at 3 T (Singh et al. 2015), ebselen treatment in healthy volunteers produced a small but significant reduction in myo-inositol in the anterior cingulate cortex but not in the occipital cortex. Interestingly, the extent of the reduction (about 4 %) was very similar in both studies, suggesting a lack of dose-response of this particular effect at the two doses of ebselen used (1800 vs 3600 mg over 24 h). In animal studies, ebselen also lowers myo-inositol in the brain presumably through its ability to inhibit IMPase (Singh et al. 2013).

The brain is thought to be relatively impermeable to the influx of inositol from plasma which means that myo-inositol in the brain needs to be synthesized from glucose-6-phosphate via myo-inositol 1-phosphate (Berridge et al. 1982). Blockade of IMPase prevents the subsequent dephosphorylation of myo-inositol 1-phosphate to inositol and in animals treated with an IMPase inhibitor such as lithium; concentrations of myo-inositol 1-phosphate are increased while those of myo-inositol are lowered (Allison et al. 1971; 1976). This effect would be expected to disrupt neurotransmission using the phosphoinositide cycle as a second messenger, and this has been postulated to be the basis of the therapeutic action of lithium (Berridge et al. 1982).

Whether, in humans, lithium lowers brain levels of myo-inositol, as measured by MRS, is controversial (Silverstone et al. 1996; Davanzo et al. 2001; Machado-Vieira et al. 2015). However, the change in myo-inositol concentration we identified in both our MRS studies of ebselen is small and apparently shows some regional specificity. If the same applies to lithium treatment, it might make detection of this effect difficult. Another issue is that as well as its role in the phosphoinositide cycle and second messenger signaling, a pool of free myo-inositol is present in astroctyes where it appears to function as an osmolyte (Brand et al. 1993). Thus, while our findings suggest that ebselen inhibits IMPase in humans, further work will be needed to demonstrate that this effect has functional consequences for neurotransmission linked to the phosphoinositide cycle. In animals, for example, ebselen treatment inhibits behavioral responses mediated by 5-HT_2A_ and 5-HT_2C_, receptors, both of which employ the phosphoinositide cycle as second messengers (Singh et al. 2013; Antoniadou et al. 2015).

The finding that ebselen lowers GSH was unexpected because ebselen was developed as a glutathione peroxidase (GPx) mimetic which should facilitate the reduction of oxidative species (Azad and Tomar 2014). Animal studies suggest that ebselen has anti-inflammatory properties in a variety of models, and an in vitro study of simulated neuronal ischemia reported that ebselen treatment resulted in increased glutathione levels and improved neuronal viability (Pawlas and Malecki 2007). Oxidative stress has been suggested to be relevant to the development of schizophrenia, and Cabungcal and colleagues (2014) have shown that ebselen administered during adolescence reversed subsequent behavioral deficits in an animal model of schizophrenia. In patients with bipolar disorder, glutathione levels are reportedly lower in both plasma and post mortem brain tissue from the frontal cortex (Rosa et al. 2014; Gawryluk et al. 2011), showing the possible importance of oxidative stress in the pathophysiology of this condition.

The function of GPx is to catalyze the conversion of reactive oxidative species using reduced GSH as a substrate; this results in the conversion of GSH to glutathione disulfide (GSSG). In the healthy brain, virtually, all GSH is present in the reduced form. Thus, although GSSG has an MRS signal distinct from that of GSH, it is estimated that under normal conditions, the contribution of GSSG to the MRS profile is negligible (Satoh and Yoshioka 2006). It is possible, however, that if the GPx-like activity produced by ebselen resulted in a significantly increased conversion of GSH to GSSG, GSH levels might be lowered when examined by MRS. Finally, GSH synthesis requires glutamate (Berk et al. 2008) and so the effect of ebselen to lower glutamate (see below) might have played a role in decreasing GSH concentration. However, the change in GSH that we saw with ebselen was small, and not predicted, and may represent a chance finding,

We also found that ebselen lowered Glx and its two major components, glutamate and glutamine, following ebselen treatment. This might indicate reduced activity at glutamate synapses, which would be of interest in view of the proposed role of ebselen in neuroprotection (Azad and Tomar 2014). For example, ebselen decreased glutamate release in rat brain synaptosomes and protected cerebellar granule cells from glutamate-induced excitotoxicity (Porciúncula et al. 2001; Nogueira et al. 2002)

Ebselen potently inhibits, glutaminase (Ki 15 nM), an enzyme that plays a key role in converting glutamine to glutamate; therefore, inhibition of glutaminase by ebselen would be expected to lower glutamate levels (Thomas et al. 2013). Whether this could also lead to lower levels of glutamine is unclear. However, because glutamine is derived from synaptically released glutamate which has been taken up by glia, it is possible that if less glutamate were available for release, levels of glutamine would fall as a consequence (Yüksel and Öngür 2010). Yüksel and Öngür (2010) suggest that Glx can be considered as representing the total amount of glutamate available for synaptic and metabolic activities, and it appears that ebselen treatment significantly diminishes this pool.

The ability of ebselen to lower indices of glutamate activity is of interest in view of the reported increase in Glx in patients with bipolar disorder (Gigante et al. 2012). This is in striking contrast to unipolar depressed patients where Glx levels in anterior brain regions tend to be decreased relative to healthy controls (Luykx et al. 2012). Indeed, it has been suggested that Glx levels, as measured by MRS, might distinguish bipolar from unipolar depression (Taylor 2014). It also suggests that ebselen might be useful in the treatment of bipolar depression which is often refractory to current medications (Vázquez et al. 2014). The effect of lithium treatment on MRS glutamate levels has been little studied, but a recent longitudinal investigation by Machado-Vieira et al. (2015) in bipolar depressed patients reported an increase in glutamate and Glx in the anterior cingulate cortex after 6 weeks lithium treatment. This suggests a striking difference between lithium and ebselen in their effect on glutamatergic mechanisms.

As in our previous study, in contrast to the effects of ebselen on brain neurochemicals in the anterior cingulate cortex, we found no changes in the occipital cortex. At first sight, this is puzzling because one might expect effects of a drug such as ebselen, which targets a second messenger system linked to several different neurotransmitters, to be manifested widely in the brain. We suggest two possible explanations. First, the effect of ebeselen on levels of myo-inositol, for example, might depend on the amount of phosphinositol-linked neurotransmission in particular brain regions and be more obvious in regions where such second messenger systems are present in high concentration. Second, the effects of ebselen might be more readily detectable when neurons in the voxel under study are in a state of activation. In this context, our MRS measures were made when participants were lying at rest inside the MR camera with their eyes closed. The activity of the occipital cortex in this situation would be expected to be low. However, anterior brain regions with their role in cognition would probably be more active, particularly if they form part of the default mode network which, in some studies, is the case for the anterior cingulate cortex (Greicius et al. 2003; Sheline et al. 2009).

A criticism of our study is that we did not apply statistical correction for the number of comparisons made in the MRS data. However, we did employ prior multivariate ANOVA, which in the anterior cingulate cortex showed a significant main effect of ebselen treatment and a treatment by metabolite interaction. Moreover, the decrease in inositol following ebselen was predicted both on theoretical grounds and from our previous study. Finally, the decrease in Glx in the anterior cingulate cortex, although modest in extent, was highly significant. However, replication of these effects, perhaps in a patient group, is clearly important. Another methodological shortcoming, which could be addressed in future work, is that we did not control for stage of the menstrual cycle in the female participants in the study.

In conclusion, we have confirmed that ebselen decreases myo-inositol concentration in the human brain indicating functional blockade of IMPase at the doses employed. Consistent with its reported inhibitory action on glutaminase, ebselen also lowers indices of glutamate activity. Both these actions suggest a potential use for ebselen in the treatment of bipolar disorder.
